# Once versus twice daily antihypertensive medications for the control of nocturnal blood pressure: a comparative study

**DOI:** 10.1186/s43044-020-00045-5

**Published:** 2020-03-04

**Authors:** Ghada Youssef, Sherif Nagy, Ahmed El-gengehe, Magdy Abdel Hamid, Amr Abdel Aal

**Affiliations:** 1 Cairo University, Faculty of Medicine, Kasr Al Ainy Hospitals, Kasr Al Ainy Street, Cairo, Egypt; 2 Helwan University, Faculty of Medicine, Cairo, Egypt

**Keywords:** BP variability, ABPM, Dipping, Antihypertensive drugs

## Abstract

**Background:**

Blood pressure (BP) shows short-term variability within the 24 h, which can only be assessed with 24-h ambulatory blood pressure monitoring (ABPM). It is of utmost importance to control BP throughout the night to reduce incidence of hypertension complications. The purpose of this study is to evaluate the effect of timing and frequency of antihypertensive medications on the average nighttime and 24-h blood pressure control.

**Results:**

The study enrolled 199 hypertensive patients with controlled office blood pressure; 135 (67.8%) patients were on once daily antihypertensive medication (group 1) while 64 (32.2%) patients were on twice daily doses (group 2). The mean office SBP was 128.7 ± 7.8 mmHg in group 1 vs 129.6 ± 6.6 mmHg in group 2, (*p* = 0.421). ABPM readings for both groups were as follows: mean daytime SBP was 125.4 ± 11.6 mmHg vs 130.1 ± 12.9, *p* = 0.011; mean nighttime SBP was 117.0 ± 12.4 mmHg vs 123.1 ± 13.9 mmHg, *p* = 0.002, and mean 24-h SBP was 122.7 ± 10.6 mmHg vs 127.5 ± 12.0, *p* = 0.005. The prevalence of non-dipping was 68.9% in group 1 vs 70.3% in group 2 patients, *p* = 0.8 (the mean dipping ratio was 0.93 ± 0.08 in group 1 vs 0.95 ± 0.07 in group 2, *p* = 0.198). The prevalence of masked hypertension was higher in group 2 (28.1% vs 43.8%, *p* = 0.029).

**Conclusion:**

Taking an extra antihypertensive pill at night did not show a decrease in the nighttime or the average 24H blood pressure in hypertensive patients with controlled office BP. On the contrary, patients who used twice daily antihypertensive medications seem to have higher nighttime and 24-h SBP, although the dipping ratio was comparable in both groups.

## Background

Hypertension (HTN) contributes adversely to cardiovascular morbidity and mortality, and treating its complications constitutes an economic burden on both developing and developed countries. Achieving guidelines-recommended blood pressure (BP) targets is of an extreme importance to reduce the cardiovascular burden worldwide [1, 2].

Despite its many drawbacks, office BP measurement is still considered the golden standard for diagnosing HTN and for determining the threshold of initiation and follow-up of anti-hypertensive medications. Many studies showed that data provided by ambulatory blood pressure (ABPM) as nighttime blood pressure and dipping status are stronger predictors of cardiovascular outcome than office BP [3]. Because of its cost and lack of availability in many health centers, ABPM cannot yet replace the office BP measurements, so it is reserved to situations where office BP alone cannot explain the clinical circumstances of the patient.

The frequency of daily administration of anti-hypertensive medications may influence the adequacy of the 24-h (24H) BP control which is the ultimate goal when approaching hypertensive patients. The exact mechanism of such phenomenon is not fully understood but may be related to several factors as adherence and compliance to prescribed drugs and change in efficacy of therapeutic coverage when various drugs are combined [4].

The aim of this study is to determine the effect of the frequency of daily doses of antihypertensive drugs on ABPM nocturnal BP readings in patients with controlled office BP.

## Methods

This is a prospective, non-randomized, observational, cross sectional study in which 199 hypertensive patients were enrolled from the Specialized HTN clinics (SHCs) at two university hospitals. Inclusion criteria included hypertensive patients with controlled office blood pressure readings (< 140/90 mmHg and < 140/85 mmHg for diabetic patients, in at least two office visits, 1 month apart) and on regular antihypertensive medications regime [5]. Exclusion criteria included secondary hypertension, acute myocardial infarction, significant valvular heart disease, decompensated heart failure (New York Heart Association class III and IV), and pregnant ladies.

The study protocol was approved by the local institutional ethics committee. A detailed, written, informed consent was taken from all the patients. Patients had full clinical evaluation including cardiovascular risk factors assessment, e.g., history of diabetes mellitus, smoking history and duration, dyslipidemia, family history of cardiovascular (CV) risk factors, and current antihypertensive drugs (class, dosage, and dosage frequency). Compliance to antihypertensive drugs was assessed by patient self-reporting, and a cutoff point of ≥ 80% adherence to prescribed medications was used for definition of patient’s compliance and non-compliance [6]. Examination included assessment of the body mass index (BMI) (Obesity is defined as BMI > 30 Kg/m2) and supine heart rate.

We recorded office blood pressure using a digital fully automated device (Omron-6 automated device) [7]. Patients were allowed to rest for 3–5 min before measurement. Three readings were taken, 1–3 min apart, and the average of the last two readings was reported. Standing BP was measured after asking the patient to stand for 2 min without support. Postural hypotension is diagnosed when there is a drop of SBP > 20 mmHg and/or drop of DBP > 10 mmHg on attempting the standing position.

Routine laboratory workup included urinalysis, serum creatinine, hemoglobin level, serum potassium, total cholesterol, low density lipoprotein, high density lipoprotein, triglycerides, fasting blood glucose, and serum uric acid. Patients underwent fundus examination to determine clinically significant hypertensive retinopathy (≥ grade II). Patients had performed albumin creatinine (A/C) ratio if there was an evidence of proteinuria in urinalysis. Abnormal A/C ratio (defined as having albuminuria above 30 mg/dl) was used as a marker of target organ damage [8]. Diabetes was defined as a fasting glucose level ≥ 126 mg/dL on at least two occasions ≥ 3 months apart in patients not known to be diabetics or prescription of oral hypoglycemic or subcutaneous insulin [9]. Chronic renal disease was diagnosed when estimated glomerular filtration rate is < 60 mL/min/1.73m^2^, albuminuria or both on at least two occasions ≥ 3months apart [10].

Patients underwent a standard 12-lead ECG to identify left ventricular hypertrophy (LVH) using Sokolow’s established criteria for LVH diagnosis [11] and to detect other abnormalities as arrhythmias, ischemic heart disease, and conduction defects.

Target organ damage (TOD) was defined when LVH, carotid bruit, more than grade II hypertensive retinopathy, peripheral arterial disease, and clinical CVD (coronary heart disease, congestive heart failure) were diagnosed, using the appropriate investigations.

Patients underwent 24H ABPM, recorded from their non-dominant arm using Holter system Model DMS300-4A8 with the device set to measure BP every 30 min in daytime and every hour during the night, according to the preset patient’s sleep cycle. Patients sustained their normal daily routines and were instructed to remain still during BP measurement. Average day, night, and 24H BP and pulse rates were recorded. Dipping (i.e., nocturnal blood pressure fall) is calculated as the ratio between mean nighttime to mean daytime systolic BP. According to the ratios obtained, dipping has been categorized into four categories: (a) normal dipping (ratio = 0.8–0.9), (b) no dipping (ratio = 0.9–1), (c) reverse dipping (ratio = ˃ 1), and (d) extreme dipping (ratio < 0.8). Non-dippers were defined as patients with no or reverse dipping [8].

Masked uncontrolled hypertension (MUCH) was defined when the average daytime, nighttime, or 24H BP readings were elevated in the presence of well-controlled office BP measurements. Hypertension is defined as been controlled when the mean daytime reading is < 135/85 mmHg, mean nighttime reading is < 120/70 mmHg, and mean 24H reading is < 130/80 mmHg.

Valid ABPM recordings had to fulfill a series of pre-established criteria, including successful recording of more than 80% of systolic BP (SBP) and diastolic BP (DBP) during both the day- and nighttime periods, with at least one BP measurement per hour.

### Statistical analysis

Quantitative variables were expressed as mean and standard deviation (SD), while qualitative variables were presented as numbers and percentages. We divided the study patients into two groups; group 1 included patients using once daily antihypertensive medications, while group 2 included patients using twice daily drugs. We compared the two groups regarding demographics, risk factors, TOD, and other clinical parameters by means of chi-square/Fisher’s exact test for qualitative data, and Student’s *t* test for quantitative data. All statistical tests were 2-sided, and a *p* value of < 0.05 was considered significant. All analyses were carried out using SPSS 20.

## Results

**Fig. 1:**
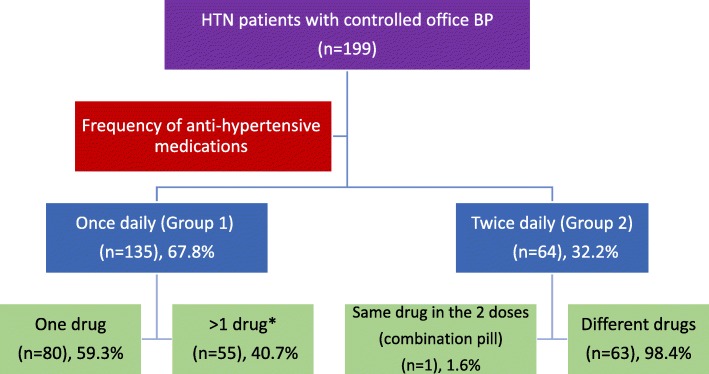
Distribution of the study population. *One combination pill or separate pills

The study included 199 patients of whom 135 (67.8%) used to take their antihypertensive drug(s) once daily (group 1), and the remaining 64 patients (32.2%) used to take the antihypertensive drug(s) twice daily (group 2) (Fig. 1).

As illustrated in Fig. 1, 80 patients received a single type of anti-hypertensive medication once per day, of whom 26 (32.5%) patients received beta blockers, 17 (21.3%) received Angiotensin receptor blockers (ARBs), 10 (12.5%) Angiotensin converting enzyme inhibitors (ACEI), 12 (15.0%) calcium channel blockers (CCBs), 12 (15.0%) diuretics, and 3 (3.8%) patients received other drugs.

The baseline clinical characteristics and laboratory findings are presented in Tables 1 and 2. Group 2 patients showed a better compliance to anti-hypertensive drugs.

**Table 1:** The baseline clinical characteristics of the study population

Variable	Total (*n* = 199) No. (%)	Group 1 (*n* = 135) No. (%)	Group 2 (*n* = 64) No. (%)	*p* value
Gender, male	94 (47.2)	55 (40.7)	39 (60.9)	**0.008**
Employment	114 (57.3)	71 (52.6)	43 (67.2)	0.052
Illiteracy	50 (25.1)	38 (28.1)	12 (18.8)	0.153
Age*, years	53.7 ± 10.1	53.4 ± 10.6	54.4 ± 9.0	0.502
Current anti-HTN medications				
Beta blockers	68 (34.2)	31 (23.0)	37 (57.8)	**< 0.001**
CCB	42 (21.1)	21 (15.6)	21 (32.8)	**0.005**
ACEI	39 (19.6)	17 (12.6)	22 (34.4)	**< 0.001**
ARBs	31 (15.6)	16 (11.9)	15 (23.4)	**0.035**
Diuretics	23 (11.6)	16 (11.9)	7 (10.9)	0.851
Combination pill	69 (34.8)	48 (35.8)	21 (32.8)	0.678
Compliance to medical treatment	187 (94)	123 (91.1)	64 (100)	**0.014**
Drug-related adverse effects	8 (4.0)	8 (5.9)	0 (0)	**0.042**
Comorbid conditions	108 (54.3)	64 (47.4)	44 (68.8)	**0.005**
DM	56 (28.1)	29 (21.5)	27 (42.2)	**0.002**
Dyslipidemia	20 (10.1)	7 (5.2)	13 (20.3)	**0.001**
Smoking	56 (28.1)	31 (23.0)	25 (39.1)	**0.018**
Cigarettes/day*	24.7 ± 5.4	24.1 ± 5.0	25.2 ± 5.7	0.488
Duration of smoking/years*	23.4 ± 9.2	22.9 ± 9.7	24.0 ± 8.7	0.651
CKD	14 (7.0)	7 (5.2)	7 (10.9)	0.138
CAD	20 (10.1)	9 (6.7)	11 (17.2)	**0.021**
CVA	9 (4.5)	6 (4.4)	3 (4.7)	0.597
PAD	6 (3.0)	3 (2.2)	3 (4.7)	0.295
Arthritis	19 (9.5)	17 (12.6)	2 (3.1)	**0.025**
Heart Failure	5 (2.5)	1 (0.7)	4 (6.2)	**0.038**
Depression/Anxiety	5 (2.5)	4 (3.0)	1 (1.6)	0.483
BPH	7 (3.5)	2 (1.5)	5 (7.8)	**0.036**
FH of HTN	133 (66.8)	86 (63.7)	47 (73.4)	0.173
FH of DM	100 (50.3)	59 (43.7)	41 (64.1)	**0.007**
FH of CAD	45 (22.6)	25 (18.5)	20 (31.2)	**0.045**
FH of SCD	26 (13.1)	10 (7.4)	16 (25.0)	**0.001**
FH of stroke	37 (18.6)	18 (13.3)	19 (29.7)	**0.006**
Abnormal fundus examination	25 (12.6)	16 (13.7)	9 (15.3)	0.777
Abnormal ECG	62 (31.2)	33 (24.4)	29 (45.3)	**0.006**
AF	4 (2.0)	2 (1.5)	2 (3.1)	0.386
LVH	24 (12.1)	12 (8.9)	12 (18.8)	**0.046**
TOD	75 (37.7)	41 (30.4)	34 (53.1)	**0.002**

*Mean ± SD

**Table 2:** Examination and laboratory findings

Variable	Total (*n* = 199), mean ± SD	Group 1 (*n* = 135), mean ± SD	Group 2 (*n* = 64), mean ± SD	*p* value
BMI, Kg/m^2^	31.4 ± 6.8	32.1 ± 7.3	29.9 ± 5.2	**0.015**
Obesity, no. (%)	95 (47.7)	69 (51.1)	26 (40.6)	0.167
Office SBP, mmHg	129.0 ± 7.4	128.7 ± 7.8	129.6 ± 6.6	0.421
Office DBP, mmHg	80.1 ± 5.2	80.3 ± 5.4	79.4 ± 4.9	0.261
Standing SBP, mmHg	131.2 ± 9.0	130.8 ± 9.3	132.1 ± 8.4	0.342
Standing DBP, mmHg	83.9 ± 7.4	83.3 ± 8.1	85.2 ± 5.7	0.055
Heart rate (supine), bpm	78.9 ± 8.6	79.1 ± 8.7	78.5 ± 8.3	0.651
Heart rate (standing), bpm	83.5 ± 9.4	84.0 ± 9.2	82.3 ± 10.0	0.240
Serum creatinine, mg/dL	1.1 ± 0.9	1.1 ± 1.0	1.1 ± 0.4	0.862
Fasting blood glucose, mg/dL	102.9 ± 28.2	101.7 ± 32.0	105.3 ± 18.1	0.415
Serum cholesterol, mg/dL	173.9 ± 31.5	176.1 ± 34.4	169.4 ± 24.1	0.179
Serum LDL, mg/dL	114.8 ± 25.2	116.6 ± 26.8	111.2 ± 21.6	0.183
Serum HDL, mg/dL	46.5 ± 8.0	47.6 ± 7.7	44.5 ± 8.3	**0.014**
Serum TG, mg/dL	157.0 ± 35.0	156.5 ± 38.6	158.1 ± 28.1	0.768
Serum uric acid, mg/dL	5.9 ± 1.4	5.9 ± 1.4	5.9 ± 1.5	0.940
Serum K, mEq/L	4.2 ± 0.4	4.2 ± 0.4	4.2 ± 0.4	0.953
Serum Hb, g/L	13.3 ± 1.1	13.3 ± 1.2	13.2 ± 1.0	0.665

Group 2 patients had a higher cardiovascular risk profile with a higher prevalence of DM, dyslipidemia, smoking, CAD and heart failure, and a higher complications rate, as evidenced by the higher prevalence of TOD. They, as well, showed a higher prevalence of positive family history of DM, coronary artery disease (CAD), sudden cardiac death (SCD), and stroke.

Office BP measurements (supine and standing) were comparable between both groups, so were the basic laboratory findings. Group 2 patients had a lower BMI, but the prevalence of obesity was the same between the two groups.

ABPM data is presented in Table 3. It shows that group 2 patients had a higher daytime (mean difference between the two groups is 4.7 mmHg), nighttime (mean difference between groups is 6.1 mmHg) as well as 24H average SBP. Diastolic BP readings were comparable between the two groups. Masked uncontrolled hypertension (MUCH) was significantly higher in group 2. Elevated nighttime BP was found in 114 (57.3%) patients, most of them were from group 1 (*n* = 70, 61.4%).

**Table 3:** Ambulatory blood pressure measurements

Variable	Total (*n*=199), mean ± SD	Group 1 (*n*=135), mean ± SD	Group 2 (*n*=64), mean ± SD	*p* value
Daytime SBP, mmHg	126.9 ± 12.2	125.4 ± 11.6	130.1 ± 12.9	**0.011**
Daytime DBP, mmHg	75.5 ± 9.4	75.3 ± 9.3	75.9 ± 9.6	0.662
Nighttime SBP, mmHg	119.0 ± 13.2	117.0 ± 12.4	123.1 ± 13.9	**0.002**
Nighttime DBP, mmHg	69.0 ± 10.3	68.4 ± 10.0	70.2 ± 10.9	0.261
24H SBP, mmHg	124.3 ± 11.3	122.7 ± 10.6	127.5 ± 12.0	**0.005**
24H DBP, mmHg	73.6 ± 8.7	73.4 ± 8.7	74.0 ± 8.8	0.646
Ambulatory HR	74.9 ± 9.8	75.0 ± 9.8	74.7 ± 10.0	0.833
Dipping ratio	0.94 ± 0.79	0.93 ± 0.08	0.95 ± 0.07	0.198
	No. (%)	No. (%)	No. (%)	*p* value
Non-dippers	138 (69.3)	93 (68.9)	45 (70.3)	0.839
MUCH				
Daytime definition	54 (27.1)	30 (22.2)	24 (37.5)	**0.024**
Nighttime definition	114 (57.3)	70 (51.9)	44 (68.8)	**0.024**
24H definition	66 (33.2)	38 (28.1)	28 (43.8)	**0.029**

**Fig. 2:**
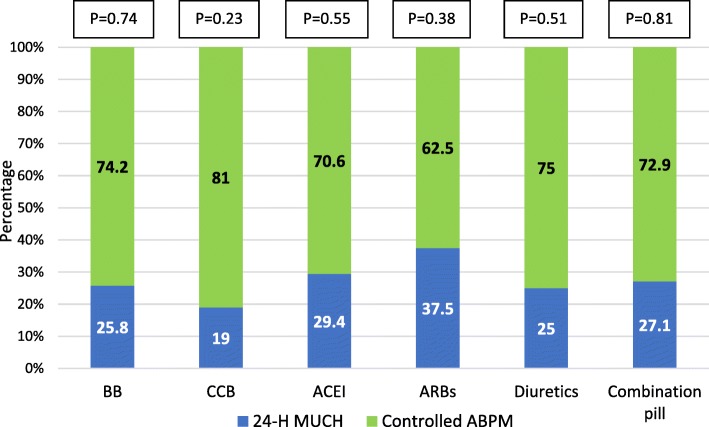
Classes of antihypertensive drugs and prevalence of 24-h MUCH in group 1 patients

Analysis of the different antihypertensive drugs taken by group 1 patients revealed absence of a significant association between the type of anti-HTN medication and the development of MUCH (Fig. 2).

## Discussion

The ultimate goal of treating hypertension is to achieve a 24H adequate blood pressure control. This cannot be detected using only office BP measurements. ABPM is the only method that can detect BP during sleep, and accordingly, the only method that can reliably define the adequacy of the 24H BP control in HTN patients [12].

ABPM measurement is an independent predictor of subsequent CV events and mortality. For every 12-mmHg increase in 24H SBP, there is a 49% increased risk of CV events, and the relative hazard per 1 mmHg for cardiovascular mortality is significantly related to the 24H SBP [12, 13]. Patients with adequate ABPM control demonstrated a lower event rate compared to those with higher blood pressure levels (0.71 events/100 person-year vs. 1.87 events/100 person-year, *p* = 0.0026) [13]. Additional prognostic information can be provided by nighttime ABPM and the dipping status of nocturnal BP with a 21% increase in the mortality risk for each 10 mmHg increase in the average nighttime SBP with bigger risks of TOD and CV events in hypertensive patients with a non-dipping pattern [14, 15].

BP regulation is characterized by physiological circadian rhythm: early morning increase in BP is known as (early morning surge, which it is strongly related to increased CV events in the morning) drastic decrease in BP during sleep in most individuals (dipping status) and a period of plateau throughout the afternoon [16]. Such circadian rhythm may be disturbed in hypertensive patients and thus must be highly considered when selecting the appropriate timing and dosing of the prescribed anti-hypertensive medications. Drugs also demonstrate a circadian-rhythm that is dependent on the pharmacokinetics and pharmacodynamics. This is known as chronokinetics which may halt the desired 24H control of HTN [17].

Achieving 24H BP control may be considerably affected by the frequency of antihypertensive medications dosing. Once daily administration is currently the preferred method of antihypertensive drug prescription given its higher edge over twice daily administration specifically related to increased patient’s adherence to simpler regimens [4]. Anti-hypertensive drugs prescribed once daily requires trough to peak ratio of at least ˃ 50% to assure a uniform 24H coverage [18]. Anti-hypertensive medications with high trough to peak ratio not only allow gradual drop of BP thus preventing adverse effects related to sudden drug action, but also normalize any blunted circadian variation in BP [4].

This study aims at demonstrating the status of nighttime BP control in HTN patients with fairly controlled office BP readings and relating nighttime control rates to the daily dosing of the antihypertensive medications.

In this study, most of the patients were taking their antihypertensive medications once/daily. Yet patients who were prescribed a twice/daily dosing of anti-hypertensive medications had a significantly higher nighttime SBP as compared to patients who used to take the drugs only once. Dipping ratio and dipping frequency were the same in the two groups; this is probably because the daytime BP of group 2 patients was also higher than the controlled values. The prevalence of MUCH (when defined using daytime, nighttime or 24H BP measurements) was higher among group 2 patients. On the other hand, more than half of the patients (57%) had elevated nighttime BP readings, and most of them were from group 1. This high prevalence of nocturnal HTN may be due to the high risk profile of the patients or the unanticipated stress accompanying the cuff inflation which awakens most patients from sleep [19].

In contrast to our study, the MAPEC study showed that patients who received their anti-hypertensive medications in two doses had lower mean 24H systolic and diastolic pressures compared to a single morning dose [20]. This discrepancy in results may be due to several factors: First, by the end of the MAPEC study only 46.8% of patients assigned to twice daily dosing were taking all the medications in the evening. Second, in our study, patients receiving twice daily dosing showed higher prevalence of CV risk factors and co morbid conditions, and finally, in the MAPEC study, the proportion of patients treated with the same medication was equal in the two treatment groups which was not the case in our study.

The high cardiovascular risk profile of group 2 patients may explain why these patients needed many drugs and accordingly why were they prescribed these many drugs twice a day. Most of the antihypertensive medications were prescribed, not only for HTN, but also for the associated comorbid conditions. This, in addition to improving compliance, explains why most of our patients were using combination pills.

The different classes of anti-hypertensive medications which is recommended to reach the BP goals may lead to uneven 24H BP control as the use of different drugs with uneven therapeutic coverage may lead to therapeutic coverage gaps [4, 5]. The study did not focus primarily on adherence to the correct timing of the prescribed drugs. Mistiming of the prescribed drugs was found to be more prevalent with twice/daily dosing compared to once/daily dosing (94% vs. 78.1 %, *p* < 0.001), and it was associated with a lower BP control (27% vs. 41%) [21].

## Limitations

This study does not reflect the general HTN population as patients were recruited from the specialized HTN clinics (SHC), and a multicenter population-based study may be required. A single ABPM recording was done, and it would have been better to repeat the ABPM to test the reproducibility of our results. Patients’ adherence to medications were assessed by self-reporting which is not the most reliable tool to confirm drug compliance.

## Conclusion

Giving two daily doses of antihypertensive medications failed to control the nighttime HTN in patients with controlled office BP. ABPM is needed in patients with high cardiovascular risk profile to detect the nighttime control of HTN and to guide the drug therapy.

## Data Availability

The datasets used and/or analyzed during the current study are available from the corresponding author on reasonable request.

## Abbreviations

A/C ratio: Albumin/creatinine ratio
ABPM: Ambulatory blood pressure monitoring
ACE: Angiotensin converting enzyme
AF: Atrial fibrillation
ARBs: Angiotensin receptor blockers
BMI: Body mass index
BP: Blood pressure
BPH: Benign prostatic hyperplasia
CAD: Coronary artery disease
CCBs: Calcium channel blocker
CKD: Chronic kidney disease
CV: Cardiovascular
CVA: Cerebrovascular disease
CVD: Cardiovascular diseases
DBP: Diastolic blood pressure
DM: Diabetes mellitus
FH: Family history
HDL: High density lipoprotein
HR: Heart rate
HTN: Hypertension
K: Potassium
LDL: Low density lipoprotein
LVH: Left ventricular hypertrophy
MUCH: Masked uncontrolled hypertension
PAD: Peripheral arterial disease
SBP: Systolic blood pressure
SCD: Sudden cardiac death
SD: Standard deviation
TG: Triglycerides
TOD: Target organ damage
